# *Hox* Genes Polymorphism Depicts Developmental Disruption of Common Sole Eggs

**DOI:** 10.1515/biol-2019-0061

**Published:** 2019-12-31

**Authors:** Menelaos Kavouras, Emmanouil E. Malandrakis, Theodoros Danis, Ewout Blom, Konstantinos Anastassiadis, Panagiota Panagiotaki, Athanasios Exadactylos

**Affiliations:** 1 Fytokou str, 38446, Volos, Greece; 2 Department of Ichthyology and Aquatic Environment, School of Agricultural Sciences, University of Thessaly, Fytokou str, Volos, Greece; 3 Wageningen Marine Research, Wageningen University & Research, IJmuiden, The Netherlands; 4 Biotechnology Centre (BIOTEC), TU Dresden, Dresden, Germany

**Keywords:** egg quality, embryonic development, *Hox* genes, polymorphism, reproduction

## Abstract

In sole aquaculture production, consistency in the quality of produced eggs throughout the year is unpredictable. *Hox* genes have a crucial role in controlling embryonic development and their genetic variation could alter the phenotype dramatically. In teleosts genome duplication led paralog *hox* genes to become diverged. Direct association of polymorphism in *hoxa1a*, *hoxa2a* & *hoxa2b* of *Solea solea* with egg viability indicates *hoxa2b* as a potential genetic marker. High Resolution Melt (HRM) analysis was carried out in 52 viable and 61 non-viable eggs collected at 54±6 hours post fertilization (hpf). Allelic and genotypic frequencies of polymorphism were analyzed and results illustrated a significantly increased risk for non-viability for minor alleles and their homozygous genotypes. Haplotype analysis demonstrated a significant recessive effect on the risk of non-viability, by increasing the odds of disrupting embryonic development up to three-fold. Phylogenetic analysis showed that the paralog genes *hoxa2a* and *hoxa2b*, are separated distinctly in two clades and presented a significant ω variation, revealing their diverged evolutionary rate.

## 1 Introduction

In aquaculture industry, *Solea solea* is considered as one of the most interesting and promising species for marine fish farming in Europe due to high market value and consumers demand. Rearing is mainly based on controlled reproduction of captive breeders [1]. The steady production of eggs either in quality and quantity all year round is necessary for successful mass production of juveniles [2, 3]. Despite the progress that has been achieved all these years on the rearing and welfare conditions of sole spawners [1, 3, 4, 5, 6, 7], manipulated broodstocks are still producing eggs which present variability both in quantity and quality, boosting the overall production cost of sole [8]. For that purpose, the availability of genetic markers to predict egg and stock viability is a very important parameter for selective breeding programs.

The factors that affect embryonic development and consequently sole egg quality include environmental conditions, physiology, nutrition and genetics [9]. *Hox* genes play a major role in controlling developmental mechanisms in all bilaterian organisms by the regionalization of the anteroposterior (A-P) axis associated with evolutionary mechanisms by introducing new body schemes in animal kingdom. They encode transcription factors involved in segmental partition and cell specificity and they were first described in fruit fly, *Drosophila melanogaster*. In teleosts, they are arranged in clusters, which vary from five to eight (Aa, Ab, Ba, Bb, Ca, Cb, Da, Db), as a result of three rounds whole genome duplication (“3R” hypothesis) and they are classified in 13 paralog groups (PG) [10, 11]. H*ox* genes are characterized by a well-conserved region called “homeobox” or “homeodomain” and also by temporal and spatial collinearity since the 3´-end genes are expressed earlier in the most anterior embryonic domains compared to their 5´ -end counterparts. The number of clusters in teleosts suggests that the duplication event took place from 110 to over 300 Mya [12, 13, 14, 15, 16, 17, 18, 19].

*Hox* gene expression is a major force either for the morphological evolution of a species, or physiological

development of embryos. Small changes regarding timing or amount of expressed genes could be critical for anteroposterior pattern [14, 18]. Strong inter and intra-species genetic variation is observed in regions that control *hox* gene expression [19, 20]. Moreover, the important characteristic of *hox* genes to auto and cross-regulate themselves is critical for their gene network expression, since a mutation in one of them could alter the phenotype dramatically [18].

*Hox* genes of PGs 1 and 2 are located at the 3´-end of *hox* clusters and are the first to be expressed in embryos in the most anterior parts and control the expression of their counterparts [21]. In mouse and *Xenopus* three PG1 genes, *hoxa1, hoxb1, and hoxd1* were identified, while in teleosts the number varies. In medaka and tetraodon three genes*, hoxa1a*, *hoxb1a*, and *hoxb1b* were found, in zebrafish *hoxc1a* was additionally described, while nine PG1 genes are present in Atlantic salmon [22]. PG1 genes are essential for correct hindbrain induction and segmentation [21]. In zebrafish, *hoxa1a* and *hoxc1a* are expressed in ventral midbrain neurons and may perform a comparable role in later neuronal patterning. On the contrary in *Fugu rubripes*, *hoxc1a* does not encode an open reading frame. *Hoxb1a* and *hoxb1b* have similar roles with *hoxb1* and *hoxa1* in mice since the first is expressed in the fourth rhombomere of hindbrain (r4) and the latter is necessary for the segmental organization of hindbrain. *Hoxb1a* and *hoxb1b* are expressed earlier compared to *hoxa1a*, which is expressed later during embryonic development. For the zebrafish *hoxc1a*, phylogenetic analysis showed that group together with mouse *hoxd1* [16]. In teleosts, the activity of *hox* PG2 genes is a key factor for the embryonic development of hindbrain and pharyngeal arches (PA), but the number (2 or 3) of PG2 genes and their expression patterns vary from species to species even to those that are closely related, implying that their function is still unclear [23, 24, 25, 26, 27, 28, 29]⁠. In tilapia and striped bass three genes of PG2, *hoxa2a*, *hoxa2b* and *hoxb2a* are present, of which *hoxa2a* is essential for PA2 development. In zebrafish, *hoxa2a* paralog is absent and both *hoxa2b*, *hoxb2a* display different functions with respect to their counterparts [25]. This is the case in which *hox* gene members of a paralog group may present diverged activity which suggests discriminative functionality among them.

It has been recorded that malformations or diverged phenotypes are inextricably correlated with *hox* allelic variations in natural populations [19, 30, 31]. Noteworthy is the fact that nucleotide polymorphism resides outside the homeodomain of *hox* genes, which remains well conserved [19, 32]. In *Takifugu rubripes*, it is reported that *hoxa* genes, after cluster duplication, present an increased mutation rate and asymmetric divergence among paralogs, with b paralogs diverging faster than their counterparts [33].

Many studies have shown that a population-based case-control design can be more powerful than a family-based study design in identifying genes predisposing both for qualitative and quantitative traits [34, 35]. In recent years, only a few studies regarding next generation sequencing for gene expression analysis in early life stages (embryonic and/ or larval stages) of *Solea solea and Solea senegalensis* were carried out. Surprisingly, only a small number of transcripts were identified as *hox* genes [36, 37]. In order to document the relation of certain *hox* gene polymorphic sites with egg viability of *Solea solea*, we recorded the polymorphism among three *hox* genes, fished and identified by using degenerated primers, cloning, and Sanger sequencing, in viable and nonviable eggs of common sole, collected at 54±6 hours post fertilization (hpf). Since the studied phenotype is binary (viable, non-viable) it makes more sense to estimate how much “risk” or “latent risk” is explained, rather than “variance” itself for complex traits. A previous work illustrated that these genes are strongly expressed during embryonic development and therefore are ideal candidates for controlling egg viability as potential genetic markers [38]. These targeted three *hox* genes that reside at the 3´-end of the *hox* clusters are *hoxa1a*, *hoxa2a*, *hoxa2b*. High Resolution Melt (HRM) analysis was applied in DNA extracted from 52 viable and 61 non-viable eggs. The two most frequently occurred non-synonymous single nucleotide polymorphisms (SNPs) and a tandem repeat polymorphism (TRP) of *hoxa1a* and *hoxa2b* genes were screened. Finally, phylogenetic analysis of PG2 genes was carried out in order to better understand the nature of the most variable *hoxa2b* gene, compared to its paralog.

## 2 Material & methods

### 2.1 Broodstock management

A common sole broodstock already acclimatized was held in the facilities of Wageningen Marine Research, IJmuiden, The Netherlands. It consisted of 38 individuals (n=38) with a female to male ratio of 21:17. For rearing and spawning conditions protocol see [38]. Spawning occurred almost every day from June until late August of 2014.

### 2.2 Egg management

Eggs were harvested from egg collectors every day. The eggs were weighted and floating eggs were separated from non-floating ones. Three conical tanks of about 80L, provided with recirculating and aeriation system were utilized for floating eggs and incubation temperature was set at 100C. All egg batches hatched at 126±6 hpf.

### 2.3 DNA extraction and cloning of hox genes

Separate pools of viable (n=10) and non-viable (n=10) eggs, of six different batches sampled at 6±6, 30±6 and 54±6 hpf, were utilized for genomic DNA extraction according to a phenol-chloroform protocol [39]. Sequences of other fish species corresponding to the following targeted hox genes, *hoxa1a*, *hoxa2a*, and *hoxa2b*, were aligned and degenerated primers were synthesized on conserved regions [38]. Sequencing of 20 clones, randomly selected, derived from the abovementioned floating (n=10) and non-floating (n=10) eggs from each gene, was performed using M13 forward and reverse primers (VBC Biotech, Vienna, Austria). Partial length of abovementioned *hox* genes were deposited in GenBank, database under accession numbers of MF163044 to MF163135. For each gene, alignment of sequences from viable (floating) and non-viable (non-floating) eggs was performed and polymorphic sites were recorded.

### 2.4 Eggs sampling and DNA extraction for HRM analysis

Floating and non-floating eggs from three of the six abovementioned egg batches were collected at 54±6 hpf in triplicates, rinsed with sterile water and stored at -240C until genomic DNA extraction. After this sampling time, the amount of non-floating eggs was zero and all collected eggs at this time were chosen for the HRM analysis. Genomic DNA extraction was performed according to a phenol-chloroform protocol with minor modifications regarding the reagents quantity [39]. DNA quantity and quality were assessed in NanoDrop ND-1000 spectrophotometer.

### 2.5 High Resolution Melt (HRM) Analysis

**Supplementary figure 1 A, B & C:**
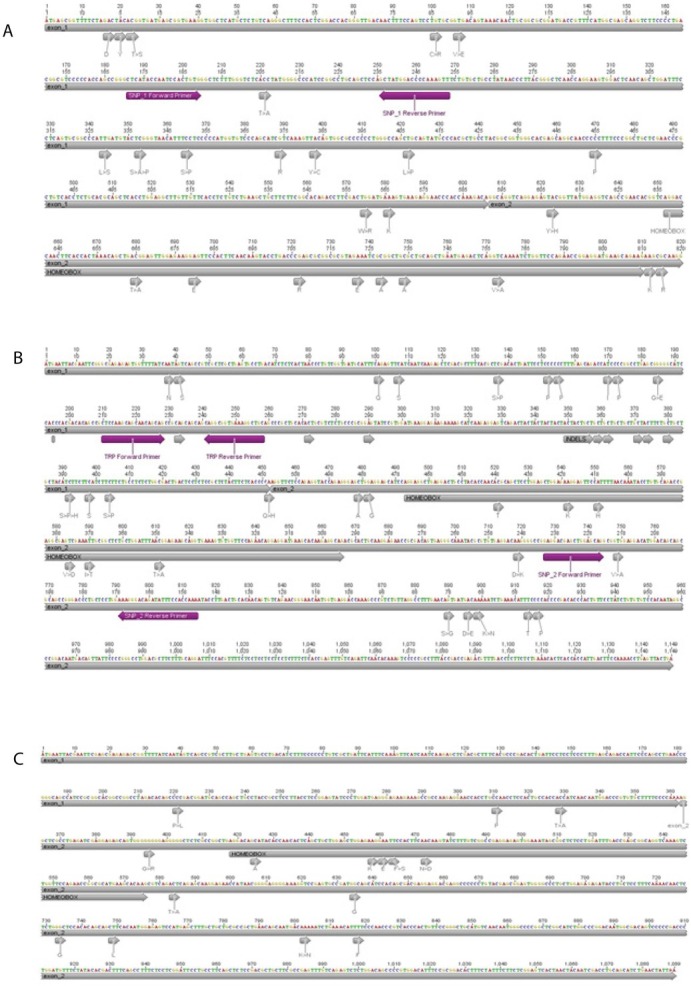
*Hoxa1a, hoxa2b* and *hoxa2a* (mRNA) polymorphism. A: *Hoxa1a* partial mRNA of *Solea solea*. SNP1 polymorphic site and the pair of primers for HRM analysis. B: *Hoxa2b* mRNA of *Solea solea*; SNP2 & TRP polymorphic sites and the pair of primers for HRM analysis are indicated. C: *Hoxa2a* mRNA of *Solea solea*, polymorphic sites are indicated. Images were created by Geneious v.4.8.5 (Drummond et al., 2009).

**Supplementary figure 2:**
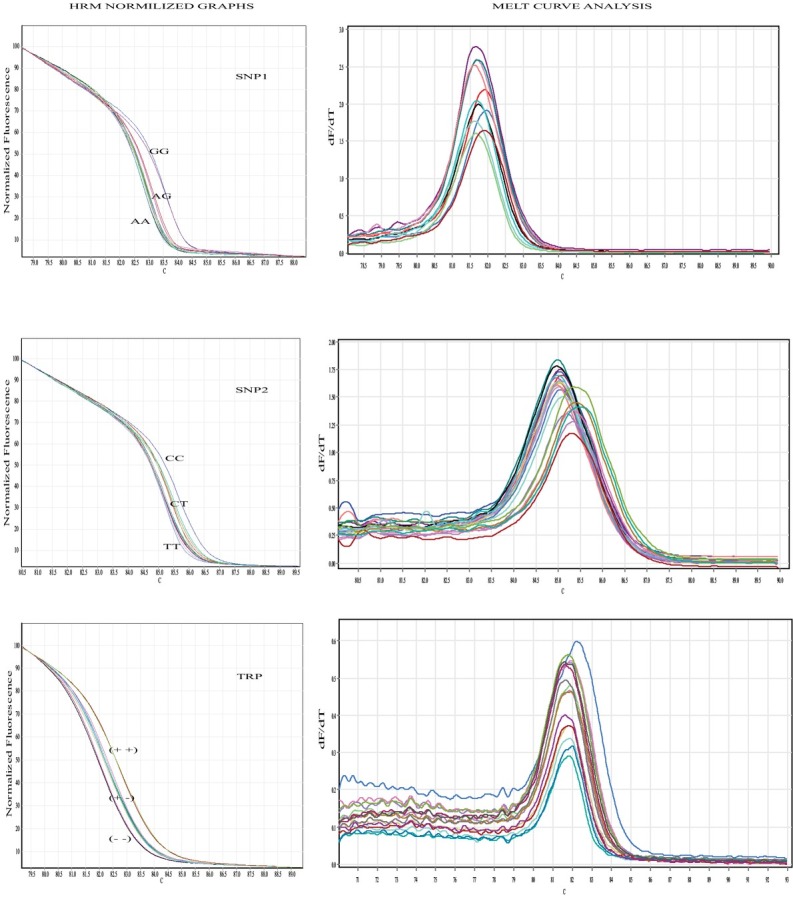
HRM normalized graphs: presenting genotypes of SNP1, SNP2 & TRP. Melt curve analysis: a single product was confirmed by uniform melting curve peaks for all three polymorphic sites (SNP1, SNP2 & TRP).

Based on the sequence alignment, the two most frequent (>15%) non-synonymous polymorphic codons and a TRP were selected, named, SNP1, SNP2, and TRP. The SNP1 resides at position 220 (A or G) of *hoxa1a* mRNA (**Supplementary fig. 1Α**), the SNP2 resides at position 749 (T or C) of *hoxa2b* mRNA (**Supplementary fig. 1Β**) and TRP which also resides at *hoxa2b* mRNA regards the deletion of the Glutamine (Q), codon (CAG), at position 232-234 (**Supplementary fig. 1Β**). For each cloned *hoxa1a* and *hoxa2b* a pair of PCR primers for each position was designed. The primer sequences and corresponding annealing temperatures are shown in **Table 1**. HRM analysis was performed on Rotor-Gene Q 5plex HRM System, Qiagen, Germany. PCR reactions with 15-25 ng of genomic DNA as template were performed in duplicates, using the two-step Type-it HRM kit (Qiagen), following manufacturer instructions in final volume of 25 μL. A single product was confirmed both by uniform melting curve peaks and single bands with expected lengths in 1.5% agarose gel electrophoresis (**Supplementary fig. 2**). PCR conditions were an initial denaturation step at 95^0^ C for 5 min, followed by a 40-cycle program (denaturation at 95^0^ C for 10 min, annealing/extension at primer specific temperature for 30 s, and HRM temperature intervals according to **Table 1**, rising at 0.1^0^ C per second). Two positive controls of each cloned haplotype already sequenced and one negative control were included in each run.

**Figure 1:**
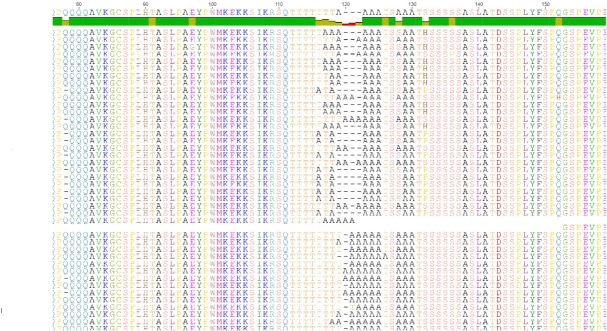
Alignment of partial HoxA2b protein sequences between non-viable and viable batch of eggs of common sole. High polymorphism of HoxA2b protein (partial) in *Solea solea*. Alignment of partial protein sequences between non-viable (1-26) and viable (29-42) batch of eggs of *Solea solea*. At the position 116-122 of consensus sequence, the indel region is evident. The presence of serine (S) exclusively in the viable batch of eggs, at the position 132 is also evident. Image of alig

**Table 1:** Annealing temperatures and size of the amplicon by the primer’s pairs, used for HRM analysis

Polymorphism	hox gene	Primer sequences (5´– 3´)	Amplicon size (bp)	T (^°^C) annealing (HRM temperature interval)
SNP1	*hoxa1a*	F: CTCATACCAATCCACTGTG	83	61,4 (78-90)
		R: AACTTTGGGGTCCATAGC		
SNP2	*hoxa2b*	F: GAGGACGAGCTGGAGCAG	88	62.8 (80-90)
		R: TTTGGTGGAAATATCTGTCCCTTT		
TRP	*hoxa2b*	F: CTCCAAGCAGCAACAGCAG	49	68 (75-90)
		R: GCAGCCTTTCACCGCCTG		

### 2.6 Taxon selection-Phylogenetic analyses

Motivated the presence of high polymorphism found in the *hoxa2b* gene, and in order to estimate the divergence between *hoxa2a* and *hoxa2b* paralogs throughout evolution, phylogenetic analysis targeted in Pleuronectiformes order and *Solea solea* species was performed. For taxon analysis 25 organisms were chosen; *Heterodontus francisci* and *Latimeria menadoensis* were the outgroup, *Lepisosteus oculatus, Polypterus bichir* and *Amia calva* as basal Actinopterygians, *Anguilla japonica* as basal teleost, and representatives of five major orders of Acanthopterygii namely, Beloniformes, Cyprinodontiformes, Perciformes, Tetraodontiformes and Pleuronectiformes were selected [40]. Selected taxa are summarized in **Table 2**.

**Table 2:** Source of the sequences used for the phylogenetic study including their accession numbers. Pleuronectiformes are shown in bold.

Lineage	Order	Scientific name	Accession number (hoxa2)	
Chondrichthyes		*Heterodontus francisci*	AF224262	
Sarcopterygii		*Latimeria menadoensis*	FJ497005	
Actinopterygii	Polypteriformes	*Polypterus bichir*	AC132195	
	Semionotiformes	*Lepisosteus occulatus*	XM_006636056	
	Amiiformes	*Amia calva*	GEUG01017022	
Teleosts			*hoxa2a*	*hoxa2b*
Elopomorpha	Anguilliformes	*Anguilla japonica*	JQ976896	JQ976897
Acanthopterygii	Beloniformes	*Oryzias latipes*	AB207976	AB207985
Acanthopterygii	Beloniformes	*Oryzias melastigma*	KX244494	KX244496
Acanthopterygii	Cyprinodontiformes	*Kryptolebias marmoratus*	KP661597	KP965864
Acanthopterygii	Perciformes	*Astatotilapia burtoni*	EF594313	EF594311
Acanthopterygii	Perciformes	*Oreochromis niloticus*	XM_00543828	FJ823133
Acanthopterygii	Tetraodontiformes	*Takifugu rubripes*	DQ481663	DQ481664
Acanthopterygii	**Pleuronectiformes**	Paralichthys olivaceus	XM_020090484	XM_020107684
Acanthopterygii	**Pleuronectiformes**	Cynoglossus semilaevis	XM_008331458	XM_008323392
Acanthopterygii	**Pleuronectiformes**	Solea solea	MF163118	MF163119

### 2.7 Sequence and Evolutionary analyses

Sequences were aligned using the Muscle algorithm [41], implemented by the MEGA7 software [42]. 24 different nucleotide substitution models were tested by MEGA7 software. The analysis involved 25 nucleotide sequences. There was a total of 1,239 positions in the final dataset. All ambiguous positions were removed for each sequence pair. Phylogeny reconstruction was inferred using the Neighbor-Joining method [43]. Bootstrap test of 2,000 replicates was applied [44]. Evolutionary analyses were conducted in MEGA7 [42]. Estimation of ω and test for adaptive evolution of the paralog genes in the Pleuronectiformes order and *Solea solea* were performed using software package PAMLX (version 1.3.1) [45].

### 2.8 Statistical methods

Alleles were assessed for Hardy-Weinberg equilibrium, in the viable group of eggs, using GenAlEx 6.5 software [46] and chi-square tests with Bonferroni corrections [47]. Test for linkage disequilibrium for all pairs of sites, using the EM algorithm, was also performed with Arlequin 3.5.2.2 software [48]. Single site analysis of allelic and genotypic frequencies was assessed by logit regression under four genetic models (additive, recessive, dominant and over-dominant). We also computed the effective sample size and statistical power using G*Power v.3.1.9.4 [49]. An effective sample size can be defined as the minimum number of samples that achieves adequate statistical power. Parameters were set for 80% power (1-β err prob=0.8) and 5% significance level (α err prob =0.05) [34]. For the haplotype analysis, logit regression was also performed for three models of inheritance (dominant, additive and recessive) in the statistical package, STATA (version 14.0) (Marchenko, Y., 2010. Haplotype analysis of case-control data. Group. https://doi.org/10.1007/978-1-4614-2245-7)

Ethical approval: The research related to animals’ use has been complied with all the relevant national regulations and institutional policies for the care and use of animals.

## 3 Results

### 3.1 Detection of polymorphism

The 3΄-end hox genes; *hoxa1a*, *hoxa2a* and *hoxa2b* are expressed early during embryonic development of the common sole and play a crucial role in this very important process. Therefore, we investigated if polymorphisms in these 3 genes are associated with egg viability in the common sole.

**Table 3** summarizes the information on the detected polymorphism at sites resulted from the alignment of sequences from viable and non-viable groups of sole eggs. Noteworthy is the fact that all three genes shared almost equal amount of synonymous and non-synonymous substitutions even in the most conserved region, the “homeobox”. The position of the polymorphic sites in the transcribed sequence of *hoxa1a, hoxa2b* and *hoxa2a* are presented in **Supplementary Figure 1A, B &C**. In the most polymorphic *hoxa2b*, as compared to the other genes, a deletion was observed regarding two sequences from the non-viable group, located at position 195 of the mRNA sequence. Another interesting observation is the presence of serine (S) at codon 131 (132 in the consensus sequence), only for the viable group of eggs. For the nonviable ones, proline (P) and histidine (H) are present in almost equal frequencies; 10/26 and 9/26, respectively. Translation of *hoxa2b* mRNA showed a glutamine (Q) rich region (-GAC- codons) consisting of eight residues of glutamine separated in the middle by the insertion of Proline (-CCG- codon). In mRNA and the translated frame this region is located at position 217-243 and 73-81, respectively. Another interesting feature of common sole *hoxa2b* gene is also the presence of poly-(T)hreonine (-ACT) and adjacent poly-(A)lanine (-GCT-) regions at position 334-372 of mRNA (112-124 in translated frame), characterized by indels (**Fig. 1**).

**Table 3:** Identified polymorphic sites of *hoxa1a, hoxa2b* and *hoxa2a* mRNA sequences, in viable (floating) and non-viable (non-floating) eggs of *Solea solea*.

	Non-synonymous substitutions and indels				Synonymous substitutions			

	Amino acid	Triplet	Position		Amino acid	Triplet	Position	
hoxa1a	T>S	ACG	22	24	D	GAC	16	18
	C>R	TGT	100	102	Y	TAC	19	21
	V>E	GTG	106	108	R	CGT	388	390
	T>A^†^	ACC	220	222	P	CCC	469	471
	L>S	TTG	343	345	K	AAA	580	582
	S>A>P S>P	TCG TCC	352 364	354 366	E^‡^ R^‡^	GAG CGA	694 721	696 723
	Y>C	TAC	397	399	E^‡^	GAA	736	738
	L>P	CTG	421	423	A^‡^	GCG	742	744
	W>R	TGG	574	576	A^‡^	GCG	748	750
	Y>H	TAC	622	624	K	AAG	811	813
	T>A^‡^	ACG	679	681	R	CGC	814	816
	V>A^‡^	GTC	772	774				

hoxa2b	S>P	TCG	136	138	N	AAT	37	39
	G>E	GGG	184	186	S	AGT	40	42
	Deletion^§^	C		195	Q	CAG	100	102
	Q^†^ (deletion)	CAG	232	234	S	TCA	106	108
	T>S	ACT	271	273	P	CCC	151	153
	E>G	GAG	289	291	P	CCC	154	156
	poly-A & poly-T	ACTACTACTGCT	346	357	I	ATC	169	171
	A>T	GCT	358	360	P	CCC	172	174
	T>A	ACT	373	375	A	GCT	361	363
	A>T	GCT	379	381	A	GCT	370	372
	S>P>H^¶^	TCT	391	393	S	TCA	397	399
	S>P	TCT	403	405	A	GCT	478	480
	Q>H	CAA	451	453	G	GGA	481	483
	V>D^‡^	GTC	583	585	T^‡^	ACG	520	522
	I>T^‡^	ATT	589	591	K^‡^	AAG	541	543
	T>A^‡^	ACG	610	612	H^‡^	CAT	550	552
	D>K	AAG	718	720	T	TTC	913	915
	V>A^†^	GTG	748	750	P	CCC	916	918
	S>G	AGT	889	891				
	D>E	GAC	895	897				
	K>N	AAA	898	900				

hoxa2a	P>L	CCC	220	222	P	CCA	310	312
	T>A	ACC	328	330	A^‡^	GCA	424	426
	G>R	GGG	394	396	K^‡^	AAG	457	459
	F>S^‡^	TTC	463	465	E^‡^	GAA	460	462
	N>D^‡^	AAC	472	474	G	GGC	634	636
	T>A	ACT	583	585	G	GGC	733	735
	K>N	AAA	802	804	L	CTT	748	750
					F	TTT	817	819

† Polymorphism (SNPs or TRP) analyzed by HRM. ^‡^ Polymorphism present in the *homeobox* region. ^§^ Deletion was observed exclusively in non-viable eggs. ^¶^ Serine was present exclusively in viable eggs. Shaded regions represent the characteristic homeobox region of *hox* genes.

### 3.2 Single site analysis

The direct relationship among the two SNPs and one TRP sites in sole embryos mortality was investigated in single site analysis. Four genetic models were assessed as well as their allelic comparison. The synopsis of SNPs & TRP description and their minor allelic and genotypic frequencies is shown in **Table 4**. In particular, among the three sites, the minor allelic of SNP2 and TRP frequencies, regarding *hoxa2b*, were significantly higher for the non-viable group (non-floating eggs). A significantly increased risk of non-viability was also observed for genotype “CC” of SNP2 (homozygous in the minor allele), under the additive, the recessive and the over-dominant genetic models. Respectively, a significantly elevated risk of mortality was also observed for TRP, under both the additive and the recessive genetic models. The best fit genetic model for both SNP2 and TRP was the recessive one with *P*<0.005 and *P*<0.01, respectively. Regarding SNP1, no significances were observed either in allelic or genotypic level.

**Table 4:** Single site analysis: Allelic and genotypic frequencies associated for all genetic models of SNPs and TRP with embryonic non-viability in case-control study. Odd ratios represent n° of folds increasing the risk of non-viability along with their significances. *P* values are after Bonferroni correction.

Polymorphism	Non	Viable,	Alleles		Additive		Dominant		Recessive		Over-dominant	
			
	Viable, n(%)	n(%)	Odd ratios	P value	Odd ratios	P value	Odd ratios	P value	Odd ratios	P value	Odd ratios	P value
			(95% Cl)		(95% Cl)		(95% CI)		(95% Cl)		(95% Cl)	
**SNPl(hoxala)**												
A	89 (78.1)	73 *(77.7)*										
G (minor)	25(21.9)	21(23.3)	1.02(0.53-1.98)	0.943								
AA (ref)	39 (68.4)	29(61.7)					1.34(0.60-3.03)	0.474				
AG	11 (19.3)	15(31.9)			1.83(0.73-4.58)	0.194					1.96(0.80-4.82)	0.142
GG	7(12.3)	3(6.4)			0.58(0.14-2.42)	0.452			0.49(0.12-2.0)	0.318		
SNP2(hoxa2b)												
T	46 (54.8)	75 (79,8)										
C (minor)	38(45.2)	19(20.2)	3.26(1.68-6.32)	0.000								
TT (ref)	20 (47.6)	29(61.7)					1.77(0.76-4.12)	0.184				
TC	6(14.3)	17(36.2)			1.95(0.66-5.82)	0.229					0.29(0.10-0.84)	0.022
CC	16(38.1)	1(2.1)			0.04(0.01-0.35)	0.003			28.31(3.6- 225,9)	0.002		
TRP(hoxa2b)												
Q(CAG)	35(47.2)	61 (62.2)										
X(---)^†^ (minor)	47(57.3)	37 (37.8)	2.21(1.22-4.03)	0.009								
QQ(ref)	9(22.0)	18(36.7)					2.06(0.81-5.29)	0.131				
QX	17(41.5)	25(51.0)			0.74(0.27-2.02)	0.551					0.68(0.29-1.57)	0.366
**XX**	15(36.5)	6(12.3)			0.20(0.06-0.69)	0.011			4.13(1.4-11.9)	0.009		

† Absence (X) of Glutamine (Q)

### 3.3 Haplotype analysis

Combined analysis of SNP2 and TRP showed that in the recessive model, the risk haplotypes alanine-glutamine (A-Q) and valine-absence of glutamine (V-X), increased significantly the risk of mortality by 1.37-fold (*P*<0.005) and 2.39 (*P*<0.0001), respectively.

The most frequently encountered haplotype found was that of A-A-X (0.41). The haplotype effect model (**Table 5**) showed a significant recessive effect on the risk of non-viability for haplotypes A-A-Q (1.71, 0.64-2.79), A-V-X (3.16, 1.67-4.66) and T-V-X (2.39, 0.66-4.12), with *P=*0.002, *P*=0.0001 and *P*=0.007, respectively (after Bonferroni correction). Interesting to note is that the A-V-X haplotype increases the odds of disrupting the embryonic development for about 3-fold times compared to the most common haplotype A-A-X. A less significant additive effect was observed for haplotypes A-A-Q and T-V-X.

**Table 5:** Haplotype analysis: Association of haplotypes with non-viability risk; *P* values are after Bonferroni correction.

Haplotypes (Aminoacids)	Additive		Dominant		Recessive	
	
	Odd ratios (95% CI)	P value	Odd ratios (95% CI)	P value	Odd ratios (95% CI)	P value
A-A-Q	1.38 (0.48-2.28)	0.003	0.11 (-0.84-1.06)	0.818	1.71 (0.64-2.79)	0.002
A-V-X^†^	1.29 (0.19-2.38)	0.021	-0.52 (-1.63-0.59)	0.359	3.16 (1.67-4.66)	0.000
T-A-Q	0.03 (-1.37-1.44)	0.964	-0.85 (-2.21-0.50)	0.218	-11.90 (-18.6-18.6)	0.990
T-V-X^1^	1.09 (-1.12-2.30)	0.079	0.59 (-1.52-0.78)	0.527	2.39 (0.66-4.12)	0.007

† Absence (X) of Glutamine (Q). A: Alanine, T: Threonine, and V: Valine

Lastly, when *hoxa1a* minor allele (G coding for Alanine) is involved in both the above-mentioned haplotypes (A-Q and V-X), the risk factor increases from 1.37 to 1.71 and from 2.39 to 3.16-fold, respectively. Accordingly, the presence of *hoxa1a* major allele (A coding for Threonine) reverses the detrimental effect of haplotype A-Q, favoring egg viability by 11.90-fold, otherwise, it has no effect on V-X haplotype.

### 3.4 Phylogenetic analysis

**Figure 2:**
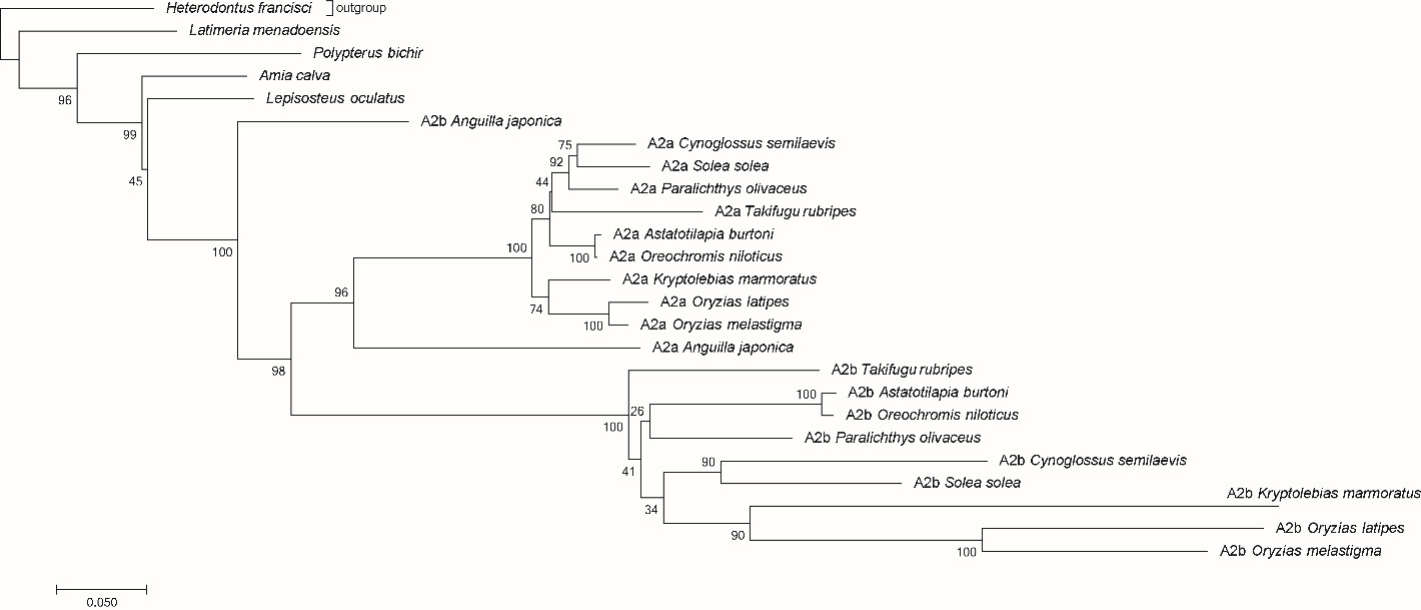
Phylogenetic tree of *hoxa2* and paralogs *hoxa2a/hoxa2b* genes. Best fit model (T92+G+I) selected by the lowest BIC value in MEGA7. Phylogeny reconstruction was inferred using the Neighbor-Joining method. The optimal tree with the sum of branch length = 2.86 is shown. The percentage of replicate trees in which the associated taxa clustered together in the bootstrap test (2,000 replicates) are shown next to the branches. The tree is drawn to scale, with branch lengths in the same units as those of the evolutionary distances used to infer the phylogenetic tree. Image of alignment was created by MEGA7 version 7.0 (Kumar et al., 2016) ⁠.

In order to justify the refined polymorphic variability of *hoxa2b* compared to its paralog *hoxa2a*, phylogenetic analysis was carried out and the evolutionary rate (ω variation) for each gene was calculated. MEGA7 software analyses showed that the best fit model with the lowest Bayesian Information Criterion (BIC) resulted to Tamura 3-parameter, using a discrete Gamma distribution (+G) with five (5) rate categories and assuming that a certain fraction of sites are evolutionary invariable (estimated value = 0.16) (T92+G+I) [50, 51]⁠ (**Figure 2)**. Bootstrap tests of 2,000 replicates were applied [45]. The rate variation among sites was modeled with a gamma distribution (shape parameter = 1.13). The differences in the composition bias among sequences were considered in evolutionary comparisons [50, 51]. This tree topology is in accordance with the known phylogeny of Actinopterygii and similar to previously performed phylogenetic analyses [33, 40]. The Acanthopterygian paralog genes (*hoxa2a* and *hoxa2b)*, are separated distinctly in two paralog clades. As for *hoxa13* gene the basal teleost *Anguilla japonica* did not follow the above clustering pattern also described by Crow et al., 2009.

Motivated by the different branch length between these paralog genes, several Likelihood Ratios statistical tests (LR) were performed regarding ω variation among sites and lineages. One – ratio model had the worst likelihood value (lnL), indicating purifying selection in overall lineages. LR tests also illustrated that **i)** the inclusion of a class of neutral sites with ω=1 is statistically justified (*P*<0.001) only under nearly neutral models, **ii)** there is a significant ω variation (*P<*0.001 after Bonferroni correction) among lineages of *hoxa2, hoxa2a* and *hoxa2b* genes with ω values equal to 0.11286, 0.12780 and 0.34535, respectively, implying a different evolutionary rate, **iii)** there is evidence of variation in ω for the branch leading to Pleuronectiformes for both paralog genes *hoxa2a* (ω=0.07) and *hoxa2b* (ω=0.36), with *P*<0.01 and 0.05 after Bonferroni correction, respectively. Improvement in the likelihood when allow ω>1, was observed only for the *hoxa2b* (*P*<0.05, after Bonferroni correction), implying that certain positions are under positive selection and **iv)** regarding *Solea solea*, significant variation in ω value was observed only for *hoxa2b* paralog (0.38, *P<0*.05 after Bonferroni correction). Evidence of variation in ω and improvement in the likelihood when allow ω>1, was detected only for the *hoxa2b*. Estimation of average evolutionary divergence over sequence pairs within groups of *hoxa2a* and *hoxa2b* (the number of base substitutions per site from averaging over all sequence pairs within each group), showed a threefold divergence, d=0.14±0.01 and d=0.40±0.02, respectively.

## 4 Discussion

Fluctuations in length of homopeptides are about 100,000 times faster than point mutations. This characteristic facilitates the morphological changes by accelerating the evolution rate of a species. This fast mutability usually adds variability to brain development and/or contributes to disease susceptibility [52].

The presence of the characteristic polymorphic homopeptide stretches of glutamine, alanine, and threonine not present in the other orthologue genes, seems to diversify *Solea solea hoxa2b* from other species. As previously mentioned, polyglutamine stretch is interrupted in the middle by a proline residue. Such stabilizing interruptions are favored by natural selection, implying an essential adaptive role [53]. Under stress, DNA methylation may induce fluctuations in homopeptide length leading to changes in gene expression and protein interactions. If stressors persist, natural selection stabilizes homopeptide by disrupting it via point mutations [52]. Polyglutamine repeats occur frequently in transcription factors and present elevated heterozygosity, as clearly observed in our results. Polyalanine and polyalanine/glutamine rich stretches are present in many repression motifs in homeodomain proteins and other transcriptional factors. These repression motifs seem to interact with the transcriptional network, regulating both gene activation and expression [54]. It has been shown that natural selection acts on protein level and is important for homopeptides’ prevalence [55]. Also, eukaryote proteins, containing homopeptide regions, exhibit distinct selective pressure characteristics which tend to be more active [56]. These polymorphic regions are source of genetic variability also known as “dynamic mutations” [57].

In the single site analysis, our results clarified the significant association between variants in *hoxa2b* gene and embryonic mortality in egg samples of *Solea solea*. Both *hoxa2b* variants, SNP2 and TRP, had a significantly higher frequency occurrence of minor alleles in the case of non-floating eggs batch, compared to the control one; an increment of mortality by a factor of 3.26 and 2.21, respectively. This fact suggests that embryos who carry Cytosine (C) and lack the triplet CAG, encoding for glutamine (Q), were significantly more likely to disrupt development, thus having an adverse effect on mortality risk. Indeed, genotypic analyses showed that in the recessive genetic model, genotype CC (coding for Alanine) and the complete absence of glutamine (Q) increases significantly the risk factor by 28.31 and 4.13-fold, respectively. To assess the joint effect of *hoxa2b* variations, combination analysis was carried out and the results indicated that there was a significant trend of increasing mortality when only one of the two minor alleles, alanine (A) or the absence of glutamine (X) is present.

A genome duplication event occurred just before the origin of teleosts [58]. Due to this fact, divergence among genes took place which led to new functions (neofunctionalization) associated with phenotypic characteristics [40, 59]. Duplicated genes relax the pressure of purifying selection in one or both counterparts, allowing the accumulation of molecular changes to occur, while the original protein functions are maintained [14, 16, 40]. As it is illustrated in the phylogenetic trees (Fig. 2), *hoxa2a* and *hoxa2b* paralogs of Acanthopterygii lineage accumulated enough changes to form two separate a and b clades. In fugu, an asymmetric divergence between paralogs was evident, with “b” paralogs diverging faster than their “a” counterparts, suggesting an increased evolutionary rate [33]. In *S. solea*, *hoxa2a* and *hoxa2b* are activated at different times during embryonic development, suggesting, that they also have distinct functional roles in regulating gene expression [38]. Due to the observed increased substitution rate, long-branches are formed [40]. Such heterogeneity in ω values between *hoxa2a* and *hoxa2b* in Acanthopterygii, Pleuronectiformes and *Solea solea*, suggests a stronger stabilizing selection for the *hoxa2a*. This heterogeneity might be due to positive selection this time or relaxed constraints in purifying selection indicating that selection acts differently upon paralog genes and among lineages [40]. However, the increased rate of evolution of *hoxa2b* this time cannot be solely explained by the presence of positive selection over the whole gene sequence, although, a few sites were detected to be under positive selection in Pleuronectiformes and *Solea solea*. By comparing the number of base substitutions per site from averaging over all sequence pairs within groups and between paralog clades an almost threefold divergence was revealed, for *hoxa2a* and *hoxa2b*. Moreover, the topology showed that rates of evolution are asymmetric between paralogs in Pleuronectiformes, *Solea solea* and the other related taxa. Duplication events usually lead to elevated evolutionary rates and divergence [40]. Although there is no clear evidence of positive selection, the significant ω ratio variation between paralogs, imply that more non-synonymous substitutions occur in *hoxa2b* and few codons might be under positive selection.

Common sole egg viability is closely related to normal embryonic development. Certain genes, especially the transcription factors, are the protagonists in the developmental scene. We showed that *hoxa2b* plays an important role in egg viability since certain types of polymorphism are significantly related to elevated risk of non-viability. *Hoxa2b* gene, presented a higher frequency of polymorphism and an elevated ω value, implying an increased evolutionary rate in respect to its paralog *hoxa2a*. Three homopeptide regions (poly-Q, poly-T and poly-A) are present only in common sole *hoxa2b*, that are usually present in other transcriptional factors involved in neural and brain development. The poly-Q region is favored by natural selection since the interruption of a proline residue has been added in the middle to stabilize its length fluctuation and consequently its mutability. This implies an important role and presumably a distinct new function that common sole *hoxa2b* acquired with respect to other species. Also, the relationship of poly-peptide tandem repeat regions with DNA methylation makes them prominent candidates for better understanding an organism’s response under stressful conditions.

Selective breeding schemes are only applied in fish species of high commercial value around the globe, such as salmon, trout, carp and tilapia. This is mainly due to the most recent scientific development on breeding aquatic living resources, in contrast with classic animal production producing high value animal proteins for humanity. Our results could assist breeders and scientists to control the role of genetic polymorphism in the early life stages of *Solea solea* development. The study focused on important targeted genes, as transcription factors, that affect normal development, thus can act as a cornerstone issue in selective breeding programs. The development of genetic markers that are related to desirable characteristics and advanced performance, could save time and money in breeding programs. With the application of molecular technology and the detection of specific markers, up-to-date know-how & know-why knowledge is introduced. Therefore, such markers could be applied synergistically with phenotypic observations during the selection scheme programs, since they can be implemented to predict egg and stock viability. Molecular markers such as *hox* gene family can be further developed to establish breeding and selection guidelines in fish broodstocks in fish that soon will be introduced in the aquaculture industry. The selection of individuals with desirable characteristics could provide competitiveness in reproductive fitness in fish farming industry.

## 5 Summary

The identification of three highly polymorphic sites that reside on *hoxa1a* and *hoxa2b* genes of *Solea solea* was labored and associated with egg viability. Analyses of polymorphism were carried out by High-Resolution Melt (HRM) in viable and non-viable eggs. Results clearly illustrated a significantly increased risk for non-viability under the recessive genetic model. Phylogenetic analysis showed that the paralog genes *hoxa2a* and *hoxa2b* of teleosts are clearly separated in two clades. Interestingly, in Solea solea, hoxa2b gene presented Poly-Q, poly-T and poly-A regions. Conclusively, the *hoxa2b* gene is being suggested as a potential genetic marker for fish breeding scheme programs.
